# Supplementary material for: A monoclinic polymorph of sodium birnessite for ultrafast and ultrastable sodium ion storage
Source: Nat Commun. 2018 Nov 30;9:5100. doi: 10.1038/s41467-018-07595-y (PMC6269426; doi:10.1038/s41467-018-07595-y)
Supplement: Supplementary file 1 — Supplementary Information [file 41467_2018_7595_MOESM1_ESM.pdf]

Supplementary Information for

Monoclinic $NaMnO_{2-y-\delta}(OH)_{2y}$ with new polymorph for ultrafast and ultrastable sodium ion storage

Xia et al.

Supplementary Figures

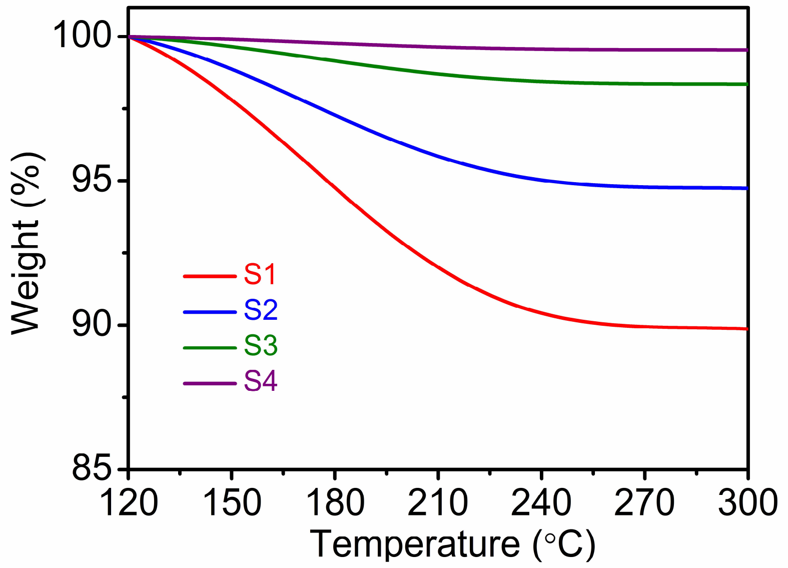

Supplementary Figure 1. TGA curves of the S1–S4 samples.

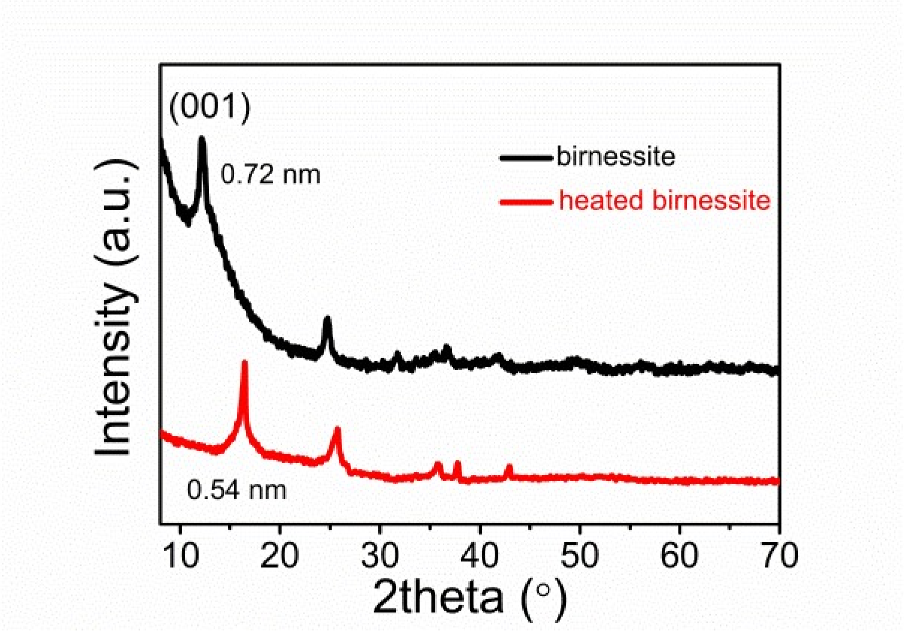

Supplementary Figure 2. XRD patterns of birnessite-$Na_xMnO_2 \cdot nH_2O$ by solution method and the heated sample.

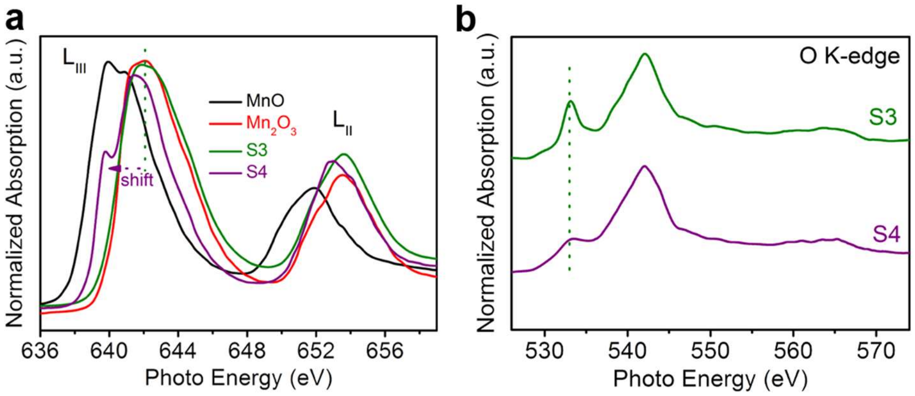

Supplementary Figure 3. a Mn $L_{II,III}$-edge and **b** O K-edge XANES spectra of the S3 and S4 samples.

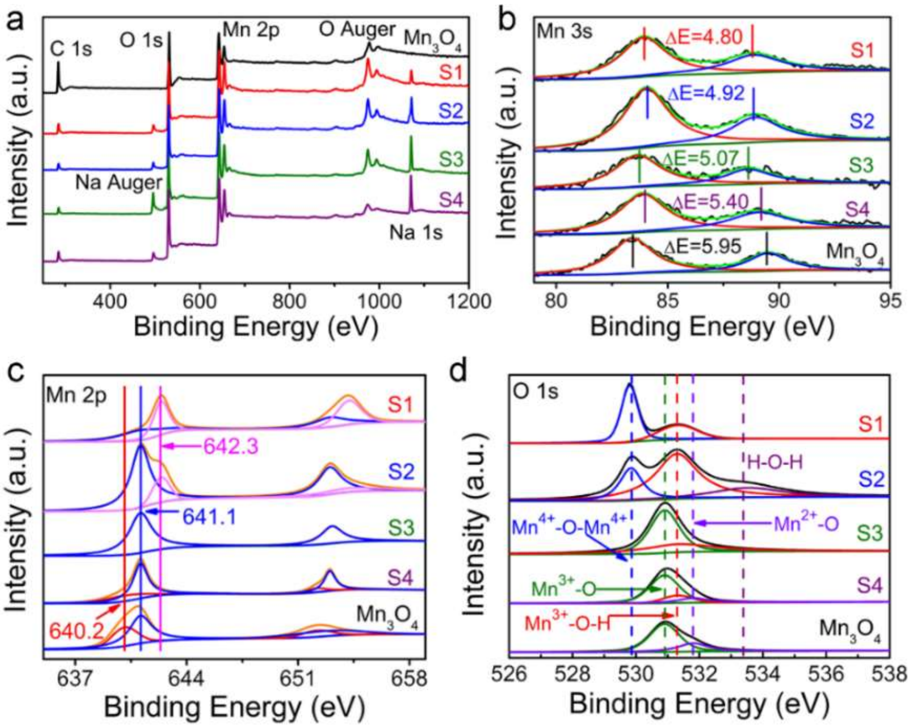

Supplementary Figure 4. **a** XPS survey scan spectra, **b** Mn 3*s* core-level spectra, **c** Mn 2*p* core-level spectra, and **d** O 1*s* core-level spectra of the Mn_3O_4 and S1–S4 samples.

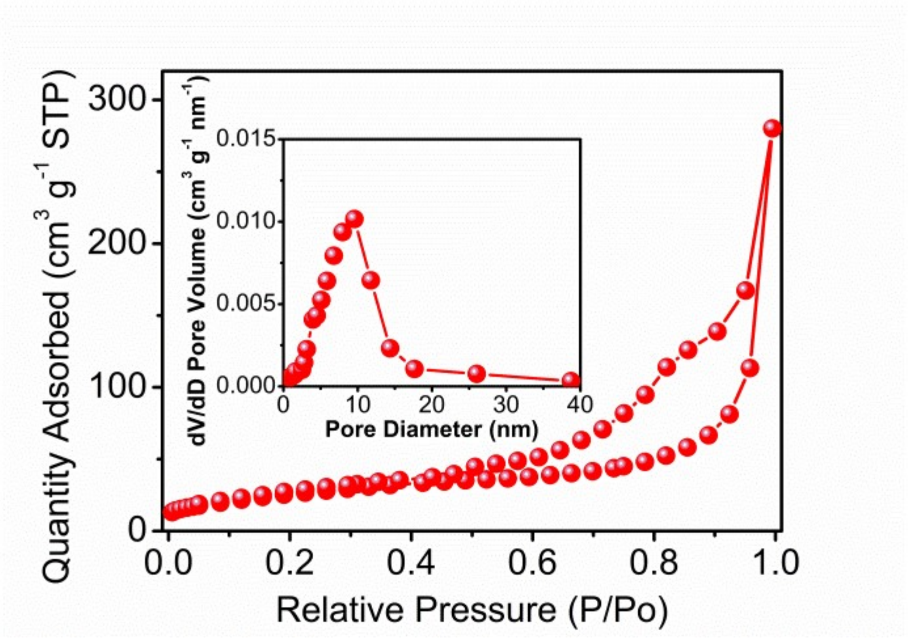

Supplementary Figure 5. Nitrogen adsorption-desorption isotherms of the S4 powdery sample (Inset is the pore-size distribution).

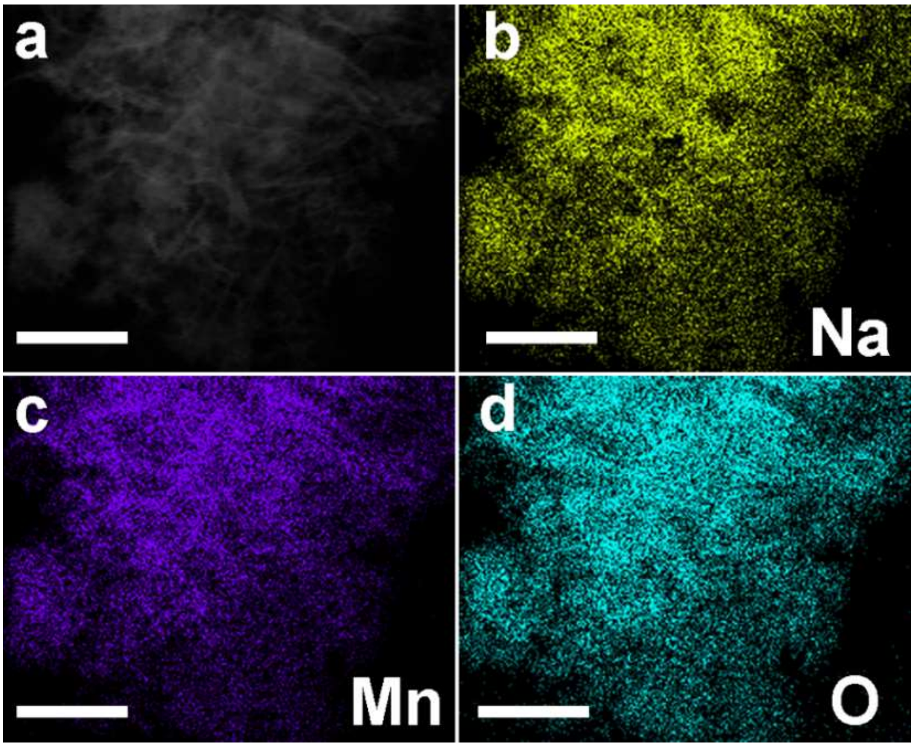

Supplementary Figure 6. The STEM image and corresponding EDS mappings of Na, Mn, and O elements for the S4 sample; Scale bar, 50 nm.

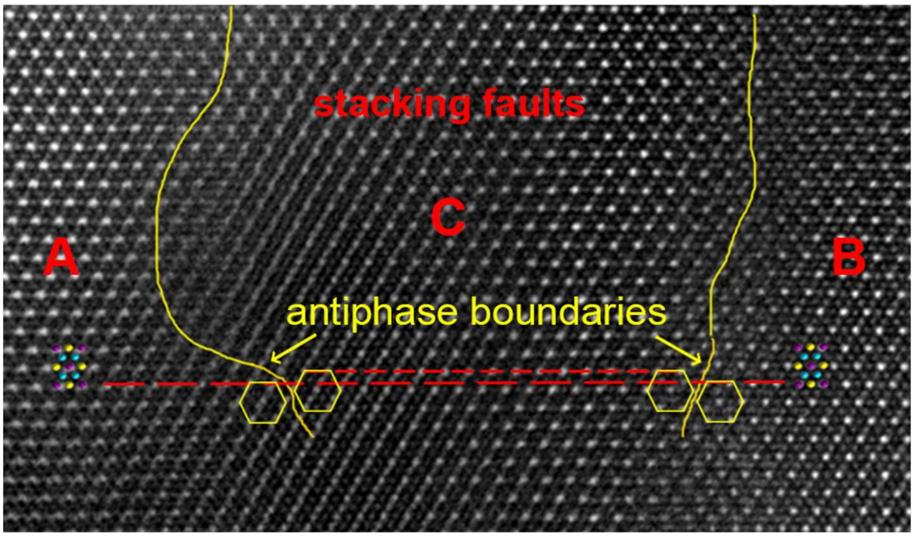

Supplementary Figure 7. HAADF-STEM image of S4 along [001] zone axis, showing the stacking faults (zone C) and anti-phase boundaries (dashed line) in H'3 (zone A and zone B have the same atomic arrangement); the purple, yellow and sky blue spheres stand for Mn, Na and OH atoms, respectively.

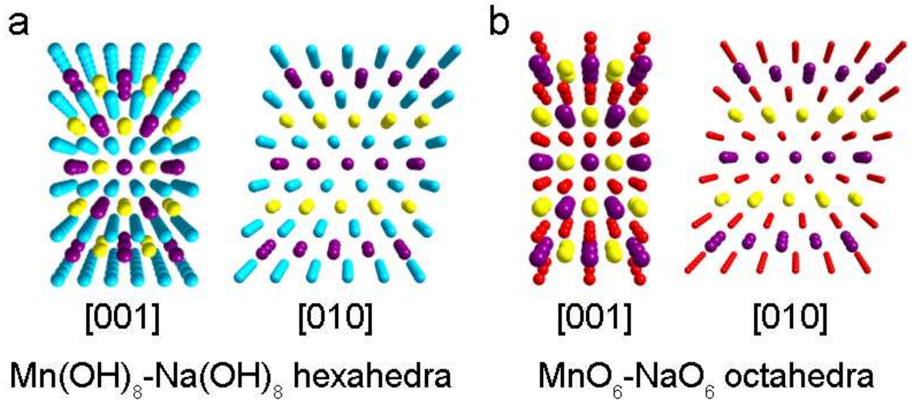

Supplementary Figure 8. Schematic atomic models of **(a)** $Mn(OH)_8$-$Na(OH)_8$ hexahedra and **(b)** MnO_6-NaO_6 octahedra along [001] and [010] zone axis, respectively; the purple, yellow, red and sky blue spheres stand for Mn, Na, O and OH atoms, respectively.

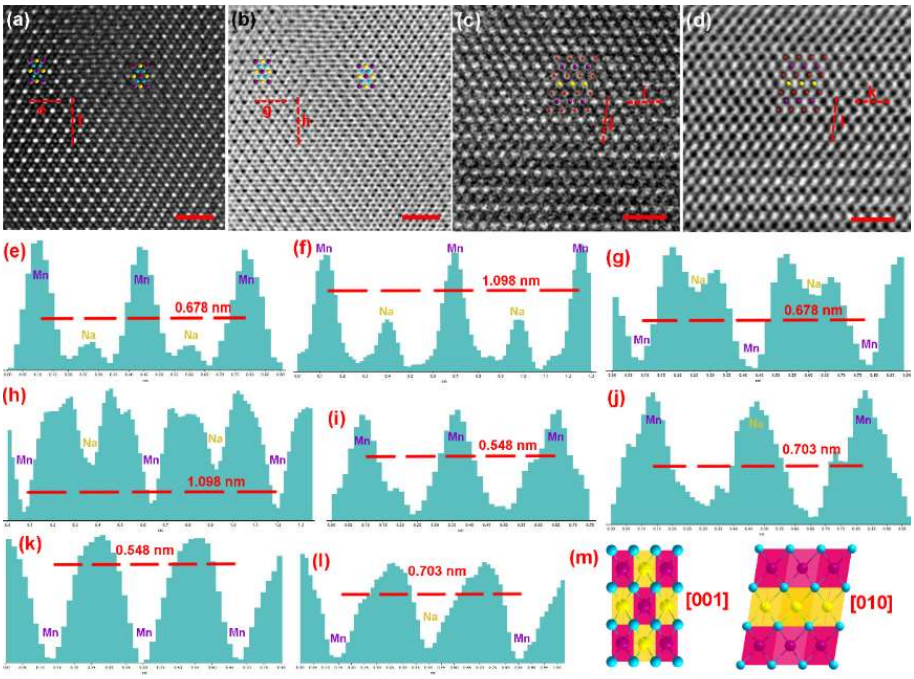

Supplementary Figure 9. (a), (b) HAADF and ABF-STEM images of H'3 phase for the S4 sample along [001] zone axis, respectively; the purple, yellow, and sky blue spheres stand for Mn, Na, and OH atoms, respectively; Scale bar, 1 nm. **(c), (d)** HAADF and ABF-STEM images of H'3 phase for the S4 sample along [010] zone axis, respectively; Scale bar, 1 nm. **(e), (f)** HAADF line profiles corresponding to the dotted lines e and f in **(a)**. **(g), (h)** ABF line profiles corresponding to the dotted lines g and h in **(b)**. **(i), (j)** HAADF line profiles corresponding to the dotted lines i and j in **(c)**. **(k), (l)** ABF line profiles corresponding to the dotted lines k and l in **(d)**. **(m)** Crystal structures of H'3 along [001] and [010] zone axis, respectively.

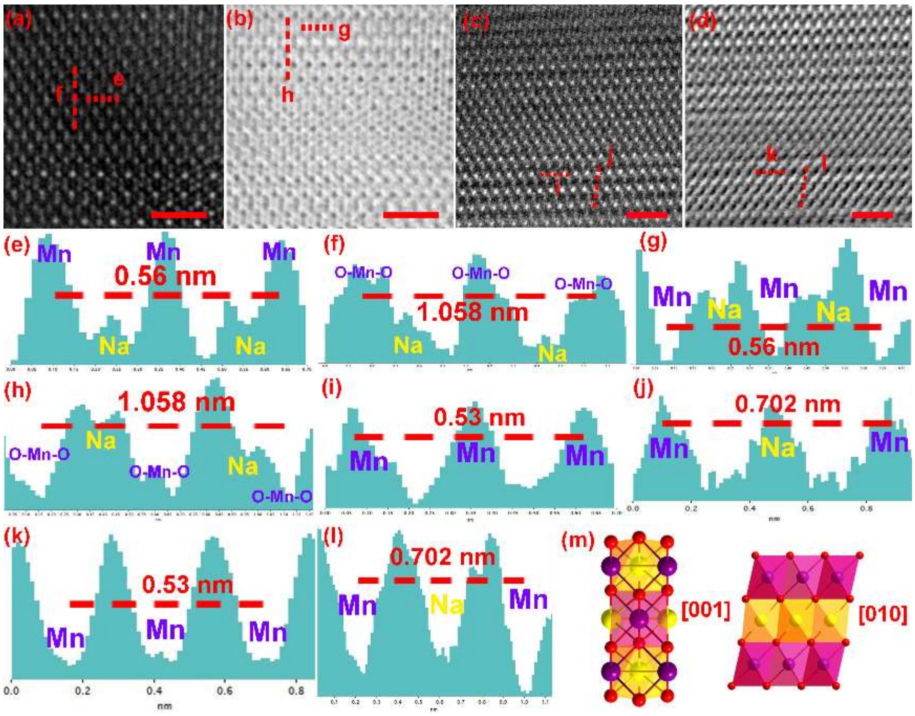

Supplementary Figure 10. (a), (b) HAADF and ABF-STEM images of O'3 phase for the S4 sample along [001] zone axis, respectively; the purple, yellow, and red spheres stand for Mn, Na, and O atoms, respectively; Scale bar, 1 nm. **(c), (d)** HAADF and ABF-STEM images of O'3 phase the S4 sample along [010] zone axis, respectively; Scale bar, 1 nm. **(e), (f)** HAADF line profiles corresponding to the dotted lines e and f in **(a)**. **(g), (h)** ABF line profiles corresponding to the dotted lines g and h in **(b)**. **(i), (j)** HAADF line profiles corresponding to the dotted lines i and j in **(c)**. **(k), (l)** ABF line profiles corresponding to dotted lines the k and l in **(d)**. **(m)** Crystal structures of O'3 along [001] and [010] zone axis, respectively.

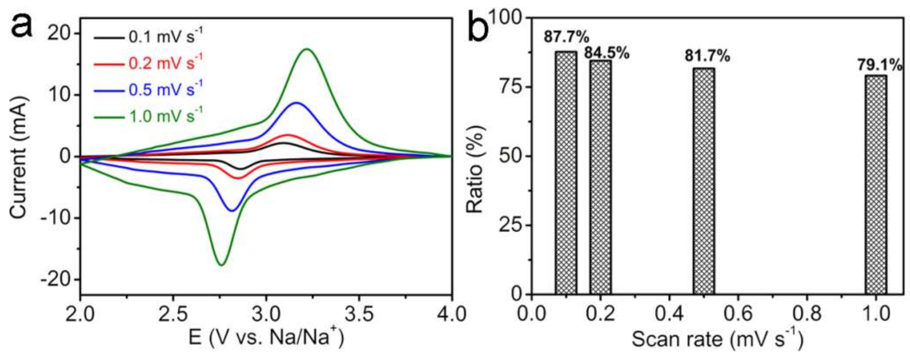

Supplementary Figure 11. a CV curves of the S4 electrode at different scan rates of 0.1-1.0 mV s^{-1}. **b** Diffusion-controlled capacity contributions at different scan rates for the S4 electrode.

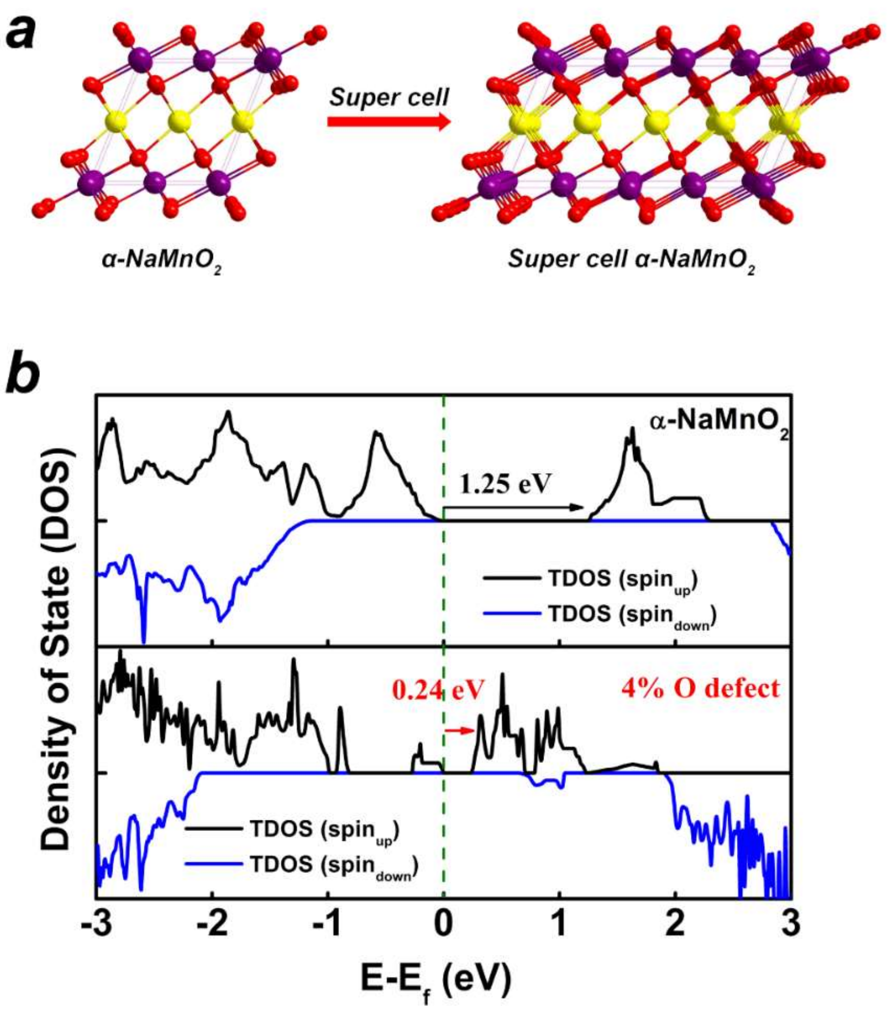

Supplementary Figure 12. a Super cell of α-$NaMnO_2$ for DFT calculations. **b** Calculated density of states of α-$NaMnO_2$ and α-$NaMnO_{2-\delta}$ with 4% oxygen vacancies; the purple, yellow, and red spheres stand for Mn, Na, and O atoms, respectively.

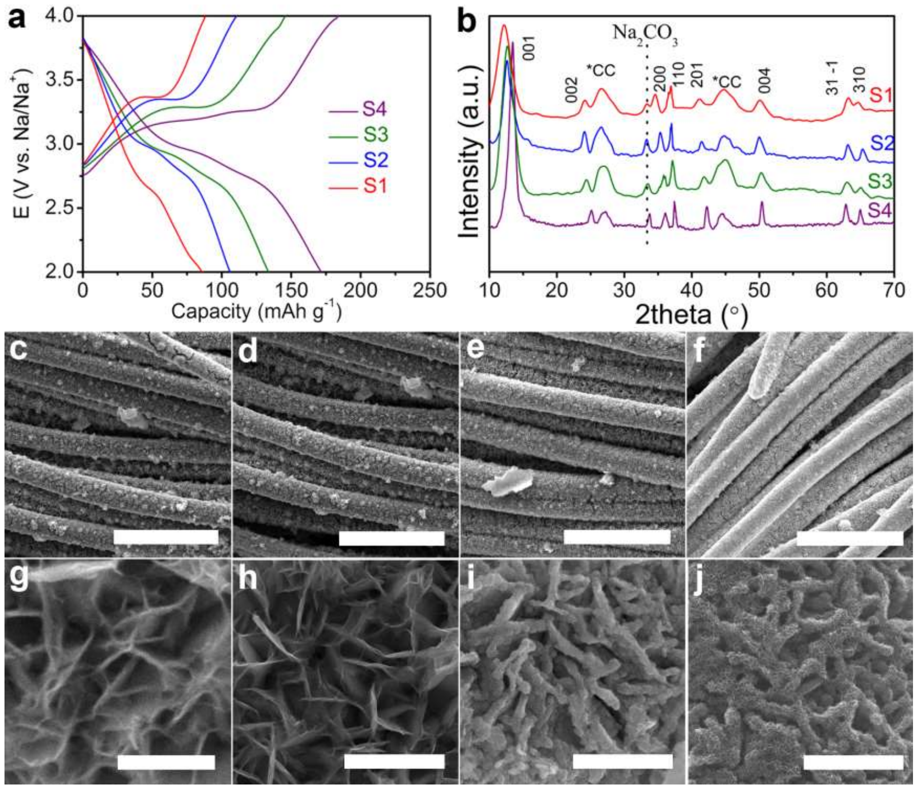

Supplementary Figure 13. **a** The 1000th charge/discharge curves of the S1–S4 electrodes at 10 C. **b** XRD patterns of the S1–S4 samples after 1000 cycles at 10 C. **c, g** FESEM images of the S1 sample after 1000 cycles at 10 C. **d, h** FESEM images of the S2 sample after 1000 cycles at 10 C. **e, i** FESEM images of the S3 sample after 1000 cycles at 10 C. **f, j** FESEM images of the S4 sample after 1000 cycles at 10 C. **c-f** Scale bar, 50 µm. **g-j** Scale bar, 2 µm.

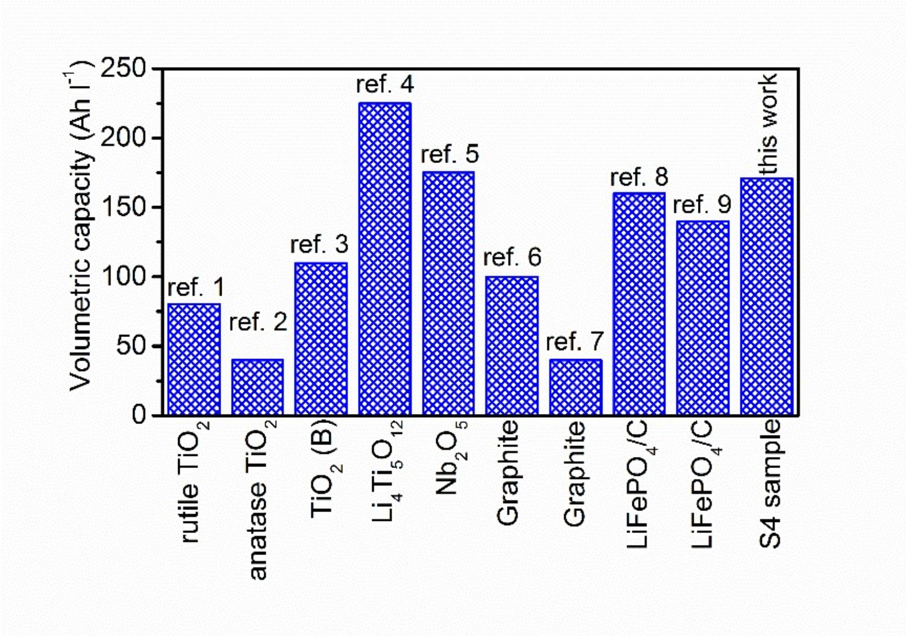

Supplementary Figure 14. The specific volumetric capacities of the S4 sample and other electrode materials in literature.

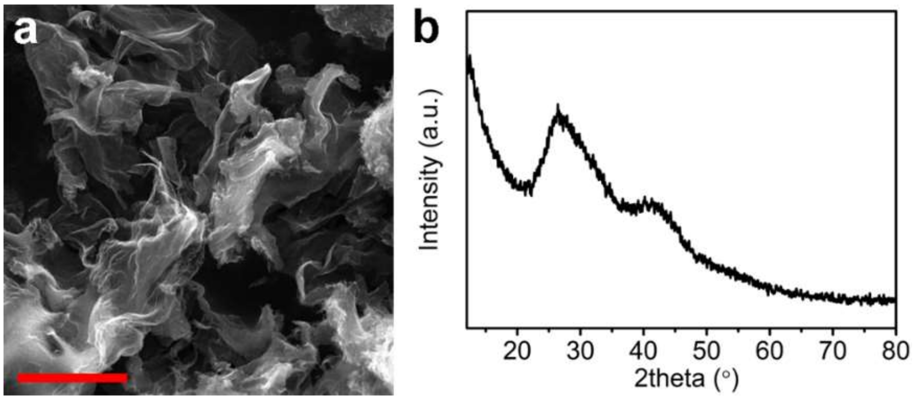

Supplementary Figure 15. a The FESEM image and **b** XRD pattern of NGS; Scale bar, 5 μm.

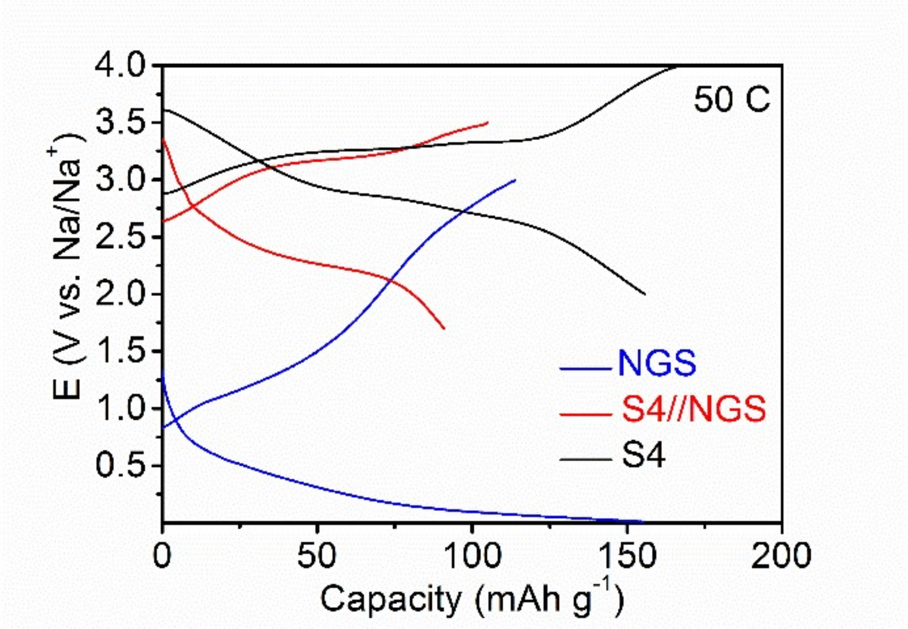

Supplementary Figure 16. Typical charge/discharge curves of the S4 cathode, the NGS anode, and the S4//NGS full cell at 50 C.

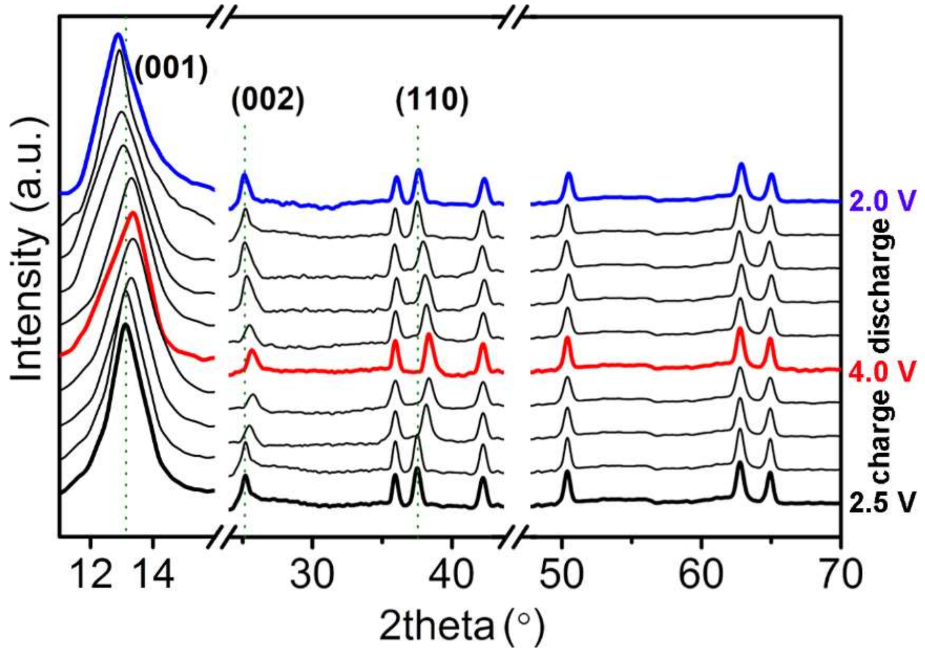

Supplementary Figure 17. Ex-situ XRD patterns of the S4 electrode collected during the first discharge/charge of the Na/ S4 half cell under a current rate of C/10 in a voltage range between 2.0 and 4.0 V.

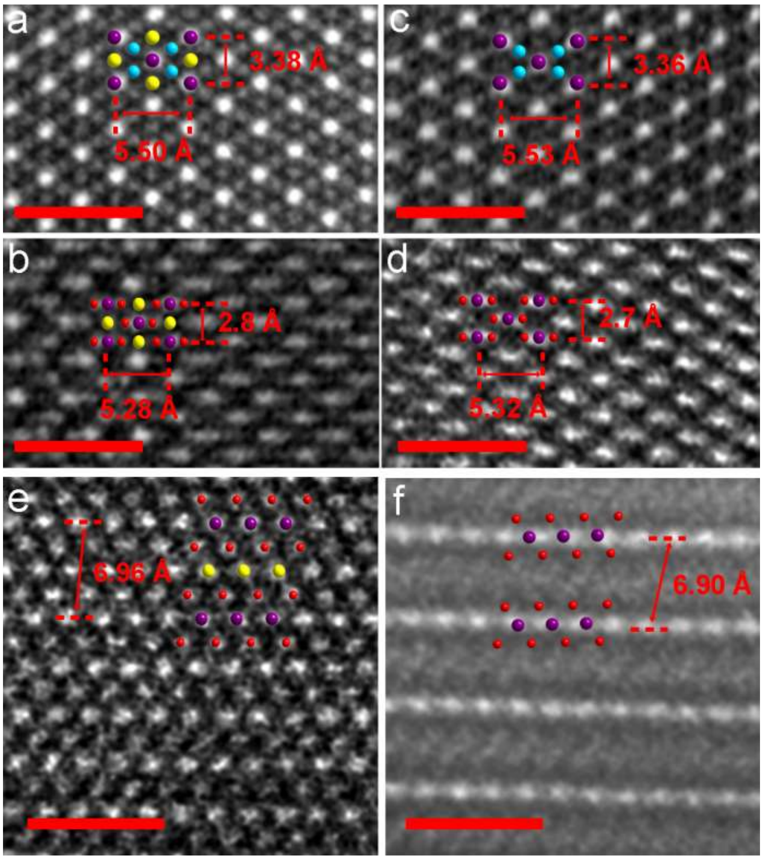

Supplementary Figure 18. a, b HAADF-STEM images for the full sodiation sample along [001] zone axis; Scale bar, 1 nm (**a,** H'3 stacking zone; **b,** O'3 stacking zone). **c, d** HAADF-STEM images for the full desodiation state of S4 sample along [001] zone axis; Scale bar, 1 nm (**c,** H'3 stacking zone; **d,** O'3 stacking zone). **e** HAADF-STEM image for the full sodiation state of S4 sample along [010] zone axis. **f** HAADF-STEM image for the full desodiation state of S4 sample along [010] zone axis.

Supplementary Tables

Supplementary Table 1. The ICP and TGA results of S1–S4 samples.

Sample	Crystal water content (*wt*%)	Na:Mn (ICP)
S1	10.11	0.46:1
S2	5.29	0.71:1
S3	1.61	1:1
S4	0.01	1:1

Supplementary Table 2. Lattice parameters of the conventional monoclinic-$NaMnO_2$ and our S4 sample from ref.7 and Supplementary Figure 5.

**Lattice parameters**	**monoclinic-$NaMnO_2$ (ref.7)**	**Our work (S4)**
Mn-O (Å), *I*	1.940	2.305
Mn-O (Å), *II*	2.390	2.402
Interlayer distance (Å)	5.334	6.900
a (Å)	5.672	5.49
b (Å)	2.856	3.39
c (Å)	5.807	7.03
β (°)	113.2	99.3

Supplementary Table 3. The crystallographic parameters of S4 from synchrotron XRD pattern.

Phase	Atom	Wyckoff symbol	*x*	*y*	*z*
Mn/NaO_6 octahedra	Na	2d	0	0.5	0.5
	Mn	2a	0	0	0
	O	4i	0.278	0	0.795
$Mn/Na(OH)_8$ hexahedra	Na	2d	0	0.5	0.5
	Mn	2a	0	0	0
	O(H)	8j	0.25	0.25	0.25
Lattice parameters (average)	a = 5.46 Å; b = 3.2 Å; c = 6.95 Å; β = 99°; space group: C2/m.				

Supplementary references

1. Hu, Y. S., Kienle, L., Guo, Y. G. & Maier, J. High lithium electroactivity of nanometer-sized rutile TiO_2. *Adv. Mater.* **18**, 1421–1426 (2006).
2. *US Research Nanomaterials* http://www.us-nano.com/home (2017). (Accessed: 16th October 2017)
3. Jiang, C., Honma, I., Kudo, T. & Zhou, H. Nanocrystalline rutile TiO_2 electrode for high-capacity and high-rate lithium storage. *Electrochem. Solid-State Lett.* **10,** A127-A129 (2007).
4. Zhang, N. *et al.* Facile preparation of nanocrystalline $Li_4Ti_5O_{12}$ and its high electrochemical performance as anode material for lithium-ion batteries. *Electrochem. Commun.* **13**, 654–656 (2011).
5. Liu, S. *et al.* Nb_2O_5 microstructures: a high-performance anode for lithium ion batteries. *Nanotechnology* **27**, 46LT01 (2016).
6. Tran, T. D., Feikert, J. H., Pekala, R. W. & Kinoshita, K. Rate effect on lithium-ion graphite electrode performance. *J. Appl. Electrochem.* **26**, 1161–1167 (1996).
7. Billaud, J., Bouville, F., Magrini, T., Villevieille, C. & Studart, A. R. Magnetically aligned graphite electrodes for high-rate performance Li-ion batteries. *Nat. Energy* **1**, 16097 (2016).
8. Wang, B., Abdulla, W. A., Wang, D. & Zhao, X. S. A three-dimensional porous $LiFePO_4$ cathode material modified with a nitrogen-doped graphene aerogel for high-power lithium ion batteries. *Energy Environ. Sci.* **8**, 869–875 (2015).

9. Chen, Z. & Dahn, J. R. Reducing carbon in $LiFePO_4$/C composite electrodes to maximize specific energy, volumetric energy, and tap density. *J. Electrochem. Soc.* **149**, A1184–A1189 (2002).
